# An updated assessment of microglia depletion: current concepts and future directions

**DOI:** 10.1186/s13041-017-0307-x

**Published:** 2017-06-19

**Authors:** Jinming Han, Robert A. Harris, Xing-Mei Zhang

**Affiliations:** Applied Immunology and Immunotherapy, Department of Clinical Neuroscience, Karolinska Institutet, Center for Molecular Medicine, Karolinska University Hospital at Solna, CMM L8:04, Karolinska Sjukhuset, S-171 76 Stockholm, Sweden

**Keywords:** Microglia, Depletion, Neuroinflammation

## Abstract

Microglia are the principal resident immune cells in the central nervous system and are believed to be versatile players in both inflammatory and physiological contexts. On the one hand, in order to safeguard the microenvironment microglia can be rapidly activated by contact with microbial products or cell debris, thereby exerting the functions of innate immunity via phagocytosis and secretion of cytokines and chemokines. Conversely, microglia can also assist in brain development, synaptic plasticity and neural repair through the production of neurotrophic factors and clearance of myelin debris. It is now well accepted that the dysfunction of microglia and microglia-induced neuroinflammation are implicated in the occurrence and progression of many neurological diseases. Although the past decade has witnessed major progress in understanding of multi-tasking microglia, what remains largely enigmatic is the relative importance of microglia at different disease stages and how microglia should be targeted for optimal therapeutic efficacy. Notably, microglia depletion through genetic targeting or pharmacological therapies can be viewed as effective tools to stimulate new microglia to repopulate the central nervous system. Microglia depletion and subsequent repopulation at defined stages in various experimental animal model disorders allow us to extend our knowledge of molecular mechanisms, thus holding promise for designing strategies to resolve neuroinflammation and promote recovery. Herein we highlight the highly plastic and diverse phenotypes of microglia and outline the lessons learned from microglia depletion approaches.

## Introduction

Microglia were first identified and named in 1919 by Pio del Rio-Hortega who is considered ‘the father of microglia’ by many neuroscientists [1]. Despite being identified 100 years ago our knowledge about microglial ontogeny and functions have only considerably advanced recently. Microglia are highly specialized and dynamic brain resident macrophages representing approximately 10% of the total cells within the adult central nervous system (CNS) [2, 3]. Microglial density varies in distinct brain locations, with a higher density being evident in grey matter in rodents [4].

Microglia are known as multi-tasking effectors and regulators in the CNS, capable of playing both pathogenic and protective roles during various phases of neurological diseases [2, 5–13]. During physiological conditions, as the first housekeeper of the microenvironment against pathogens, resting microglia are characterized by a small cell body and ramified morphology with multiple branches that actively and efficiently scan the surrounding environment, ensuring CNS homeostasis and impacting neural development [14, 15]. Upon stimulation microglia withdraw their processes, rapidly convert into a so-called amoeboid activated morphology state and release bioactive molecules, thus contributing to active inflammatory responses and combating foreign invasion at damaged sites [16, 17]. Activated microglia are thus quite plastic cells that may adopt diverse phenotypes in response to different stimuli and various microenvironmental changes [18, 19].

Newly emerging neurobiological functions of microglia are currently being investigated [20–23]. Microglia appear to influence synaptic development and connectivity, regulate other immune cells and refine the neural circuits, thereby properly determining overall brain function [24, 25]. So appropriate functioning of microglia is of major importance for CNS in both health and disease conditions.

Similarly to tissue macrophages in the periphery, microglia are also commonly subdivided into two functional categories: the classically activated pro-inflammatory M1 phenotype characterized by production of pro-inflammatory and neurotoxic mediators; and the alternatively activated M2 phenotype involved in tissue repair and remodeling characterized by secretion of anti-inflammatory mediators [26, 27]. Currently, abundant experimental evidence indicates that the dysregulation of microglia and the imbalance of these functional activation states can give rise to many autoimmune diseases [26, 28]. So targeting microglia and adjusting the balance of functional phenotypes is an attractive therapeutic strategy for inflammatory disorders [6, 29, 30]. In a previous study we have demonstrated that adoptive transfer of specifically pre-activated immunomodulatory adult microglia stimulated with a combination of interleukin (IL)-4/IL-10/transforming growth factor-β (TGF-β) could efficiently suppress the development of experimental autoimmune encephalomyelitis (EAE) in DBA/1 mice [31, 32]. However, the precise cellular involvement in this paradigm and how microglia should be specifically targeted to perform an optimal therapeutic efficacy are still poorly understood.

## Microglia origins: from the yolk sac or non-yolk sac sources?

We should first understand the origin of microglia before we discuss their functions as potential therapeutic targets. Although microglia have been studied for more than 100 years, the precise origin of microglial cells during natural development and experimental intervention has historically been controversial [33]. Some experts including Pio del Rio-Hortega himself argued that microglia originated from hematopoietic stem cells (HSCs) because of their phenotypical and morphological resemblance to peripheral tissue macrophages and monocytes. Indeed, HSCs are the founders of meningeal macrophages and monocytes. In addition, circulating monocytes can enter the brain and then differentiate into microglia-like cells [34, 35]. However, a new wave of studies has demonstrated that microglia have distinct origins and tissue-specific functions in comparison to other tissue macrophages [36–38]. In general, microglia can enter the embryonic brain and take up residence before the differentiation of other cell types. HSCs can be detected at around embryonic day 10.5 (E10.5), while F4/80^+^CD11b^+^ microglia during embryogenesis can be found as early as E8.0 and yolk sac progenitors begin to migrate to the brain at around E9.5 [39–41]. The presence of primitive microglial precursors in the developing brain earlier than definitive hematopoiesis indicates that microglia in fact exist independently of HSCs [39].

This concept has been strengthened using cutting-edge genetic fate mapping tools [37, 42]. Using this elegant tool Ginhoux et al. reported for the first time that microglia exclusively derive from progenitors outside the CNS in the yolk sac during early development, and can be constantly self-renewed throughout adult life with no input from peripheral cells in the healthy situation [37]. Additionally, microglial development does not require transcription factor Myb which is important in controlling HSC differentiation, but depends on several others such as Runx1, Pu.1 and IRF8 [43].

During development, Pu.1 is responsible for the transition from microglial precursors to the A1 state (CD45^+^C-kit^lo^CX3CR1^−^F4/80^−^), while IRF8 is essential for the maturation from the A1 to A2 state (CD45^+^C-kit^−^CX3CR1^+^F4/80^+^) [38]**.** Notably, the number of microglia in mice is sharply reduced through pharmacological blockade or genetic defects in colony-stimulating factor receptor (CSF1R), a critical factor that controls microglia proliferation [17]. Conversely, over-expression of CSF1 increases the number of microglia by promoting microglial proliferation in vivo [44]. Furthermore, IL-34 and TGF-β are also essential for microglial development [45]. A recent study presents an exciting report that human pluripotent stem cells can differentiate into microglia-like cell subsets, high concentrations of CSF-1 and IL-34 being necessary in this protocol [46, 47]. Collectively, these investigations showed that microglia arise exclusively from yolk sac in the steady state and have different tissue-specific functions.

**Fig. 1:**
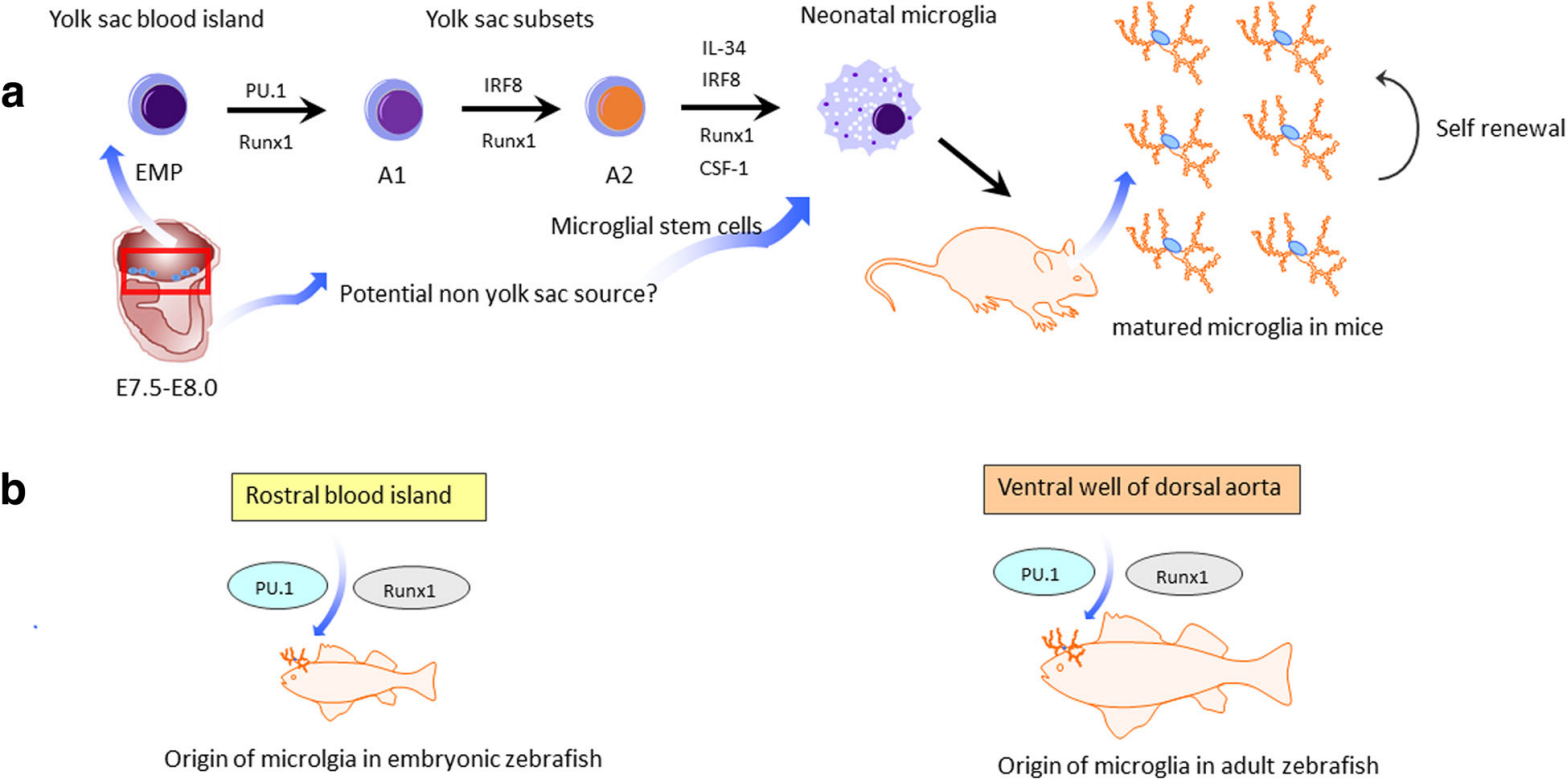
Overview of microglial ontogeny in mice and zebrafish. **a** Microglia in mice derive from the immature erythromyeloid progenitors (EMPs) outside the central nervous system in the yolk sac during around embryonic day 8.0. During development, EMPs can enter the embryonic brain and take up residence, which is regulated by factors Pu.1, IRF8 and Runx1. During further development, IL-34 and CSF-1 are needed to promote microglia proliferation. Microglia can be constantly self-renewed throughout adult life. However, whether there are potential hidden progenitors besides the yolk sac have not been identified as yet. Potential non-yolk sac sources may also give arise to microglia in mice. **b** Two different origins of microglia can be found in zebrafish. Specifically, embryonic microglia arise from rostral blood islands in zebrafish, while adult microglia originate from ventral walls of dorsal aorta instead.

However, later studies challenged this single source viewpoint by using temporal-spatial resolution fate mapping [48]. This study indicated that two different origins of microglia could be identified in zebrafish. Specifically, it was demonstrated that embryonic microglia arise from the rostral blood island in zebrafish, while adult microglia originate from the ventral walls of the dorsal aorta instead. Although these different results may simply reflect the different animal species involved, this study raises the question as to whether there are potential hidden progenitors in mice and human that have as yet not been identified. Potential non-yolk sac sources such as fetal-liver progenitor cells may also give rise to microglia. A recent study showed that fetal-liver progenitor cells could contribute to the resident microglial pool only at early postnatal stages due to rapid depletion by apoptosis [49] (Fig. 1).

When it comes to certain conditions such as whole body irradiation and global depletion of microglia, however, the repopulation of CNS local dying or depleted microglia by bone marrow-derived macrophages that adopt a microglia-like phenotype has also been reported to occur [34]. Following direct body irradiation, circulating Ly-6C^hi^CCR2^+^ monocytes were shown to traffic to the brain and then to differentiate into microglia, and could thus serve as direct precursors of microglia [34]. Subsequent studies reported that global depletion of microglia by administering a CSFR1 inhibitor did not destroy the blood brain barrier, but could trigger mobilization of latent microglia progenitors expressing the stem cell marker nestin throughout the CNS, resulting in rapid repopulation within 14 days [50, 51]. Further studies are needed to reveal the precise origin and molecular mechanisms of repopulated microglia.

## How to specifically distinguish microglia from other myeloid cells?

As mentioned above, microglia in fact have distinct origins and tissue functions compared with related cell types. However, microglia share many surface markers such as F4/80, CD11b and CX3CR1 with peripheral tissue macrophages. In addition, macrophages can also infiltrate the brain in inflammatory contexts. So it is critically important to distinguish microglia from other myeloid cells in order to have a better understanding of the roles of microglia in both health and disease states.

It is generally thought that the level of CD45 on the CD11b positive population is an effective way to distinguish microglia from macrophages [52–54]. In accordance with this notion, the CD45^int^ (or CD45^low^) expressing cells in the CD11b positive population are usually considered to be microglia, while the CD45^hi^ expressing cells are commonly viewed as being blood-derived macrophages. However, this method relies on relative surface marker expression as assessed by flow cytometry and has the serious limitation that CD45 expression can change in the context of inflammation [52, 55]. More recently, generation of knock-in mice based on high microglial expression of the fractalkine receptor CX3CR1 have advanced the cellular phenotyping [56]. Nonetheless, this method also has a limitation because circulating monocytes and other myeloid cells can also express CX3CR1 [52]. Morphological features of microglia are also widely used together with immunohistological staining for ionized binding adaptor molecule-1 (Iba-1) [53].

Ongoing transcriptional studies have identified some potential markers that are selectively expressed in microglia, such as *P2RY12, Siglec-H, olfactomedin-like 3* and *Sal1*, and that thus distinguish microglia from other related cells [17, 40, 57]. Recent reports identified the transmembrane protein 119 (Tmem119) as a potential microglia-specific marker in both the mouse and human CNS [52, 58]. Tmem119 mRNA expression is specific to all microglia and is highly enriched within the CNS, while bone marrow-derived cells in the adult CNS do not express Tmem119 [52]. Consistent with mRNA expression, immunostaining indicated that Tmem119 is limited to parenchymal CX3CR1^−^GFP^+^ and Iba1^+^ cells, and was not detected in the meninges and choroid plexus. Tmem119 only specifically labeled parenchymal resident microglia, allowing for the visualization of microglia in the context of diseases and providing a clear distinction between microglia and other related myeloid cells [52, 58].

As we have previously proposed, it is function rather than form that is critical in defining myeloid cell subpopulations [59, 60]. In vitro functional responses can thus also be used as another tool to distinguish microglia from other myeloid cells. Microglia were demonstrated to respond differently to the same stimulus depending on if another stimulus precedes or follows it [61]. To discover potential discriminatory differences between microglia and other myeloid cells, researchers have exploited a total of five polarity phenotypes based on the responsiveness of microglia and macrophages to an inflammatory polarization gradient [61, 62]. Lipopolysaccharide (LPS) and IL-4 were applied alone, sequentially in a reversible fashion, or simultaneously to microglia and macrophages. The results revealed that primarily cultured microglia could not counteract the initial M1 and M2 states first induced with LPS or IL-4 (as assessed by the expression of tumor necrosis factor-α (TNF-α) and mannose receptor C, type 1 (MRC-1)) while macrophages could. One interpretation is that microglia appear less prone than peripheral macrophages to phenotypic redirection, which provides another novel way to distinguish microglia from other myeloid cells in vitro [61]. Further molecular evaluation is needed to characterize if these observations are still valid in the in vivo setting.

## Pharmacological treatment for microglia elimination

In order to specifically deplete microglia in the CNS many pharmacological strategies have been developed (Table 1). Microglia are the only type of immune cells expressing CSF-1 in the CNS under physiological conditions [56] and as described before, the survival and development of microglia critically depends on CSF-1R signaling [51, 63]. Administration of a dual CSF-1R inhibitor can thus effectively wipe out microglia without harmful effects to mice [51, 64]. By using mice that express yellow fluorescent protein under the control of the Rosa26 locus in CSF-1R expressing cells, it has been confirmed that CSF-1R inhibition can effectively deplete microglia in the CNS instead of down-regulating microglial markers [65]. Such CSF-1R inhibition has been widely used to investigate the effects of microglial depletion with a subsequent analysis of the consequences in different disease animal models**.**

**Table 1:** Overview of pharmacological microglia depletion studies

Pharmacological intervention	Efficiency	Time window	Physiological effects	References
CSF-1R inhibitor (PLX3397)	99%	21 days	has no cognitive or behavioral impairments	[63]
CSF-1R inhibitor (PLX3397)	~90%	21 days	promotes brain recovery in intracerebral hemorrhage	[66]
CSF-1R inhibitor (PLX3397)	~90%	21 days	exacerbates brain recovery in brain ischemia	[69]
CSF-1R inhibitor (PLX3397)	97%	21 days	increases infarct size and brain injury after stroke	[67]
CSF-1R inhibitor (PLX5622)	~90%	2 or 6 weeks	ameliorates radiation-induced cognitive deficits	[70]
CSF-1R inhibitor (PLX5622)	~90%	7 days	ameliorates inflammation induced by neuronal lesion	[64]
CSF-1R inhibitor (PLX5622)	~80%	28 days	prevents neuronal loss and contextual memory in Alzheimer’s	[65]
CSF-1R inhibitor (GW2580)	Not shown	6 weeks	attenuates depression-like behavior and kidney function	[71]
Liposomal clodronate	70%	2 weeks	decreases anxiety and despair behaviors throughout life	[76]
Liposomal clodronate	~80%	1 or 5 days	alters spatial learning performance and social behavior	[75]
Mac-1-saporin	50%	1 day	triggers bone marrow derived-cell infiltration into spinal cord	[68]

Microglia can be repopulated within a short time after cessation of this treatment [51]. Of note, microglia depletion could be sustained when mice received continuous treatment. This point was confirmed by recent studies showing that approximately 90% of CD11b^+^CD45^int^ microglia in mice can be depleted by administration of CSF-1R inhibitor for 21 consecutive days [66, 67]. PLX3397, a small molecule CSF-1R inhibitor, can cross the blood brain barrier and thus rapidly deplete microglia in the CNS [51]. Similarly to microglia in the brain, microglia in the spinal cord can also repopulate rapidly following depletion with Mac-1-saporin, a selective microglia immunotoxin which can induce the breakdown of the blood spinal cord barrier and the production of pro-inflammatory mediators [68].

However, contradictory findings about the effects of microglial depletion have been reported in different animal models. More specifically, microglia elimination by PLX3397 was shown to exert neuroprotective functions by preserving blood brain barrier integrity and thus reducing leukocyte infiltration into the CNS in the context of intracerebral hemorrhage [66]. By contrast, microglia elimination by PLX3397 was shown to exacerbate neuroinflammation and brain injury in the context of brain ischemia [67, 69]. Moreover, other pharmacological approaches to specifically deplete microglia have also been used recently. PLX5662 treatment, a more brain-penetrant CSF-1R inhibitor than PLX3397, can cause a rapid and significant depletion of IBA-1 and CD68 positive microglia in the CNS within 3 days [70]. Elimination of microglia by PLX5662 can ameliorate cranial radiation-induced cognitive deficits in mice, when tested by a battery of behavioral tasks 4–6 weeks after irradiation [70].

These findings, along with others, indicate that acute microglia depletion by using PLX5662 and then subsequent repopulation can resolve neuroinflammation and promote brain recovery, despite extensive neuronal loss in the hippocampus [64]. Importantly, recent studies have confirmed that GW2580, another CSF-1R inhibitor, can deplete both microglia and macrophages at the same time [71]. On the one hand, daily treatment of GW2580 can attenuate neurobehavioral deficits such as depression-like behavior in murine lupus. Conversely, GW2580 treatment can also deplete IBA^+^ macrophages within the kidney, contributing to protection of kidney function [71]. In this regard, CSF-1R inhibitors may not be specific for microglia in the CNS. Indeed, both tissue macrophages and microglia express this receptor, and treatment with CSF-1R inhibitor can also inhibit other kinases and suppress immune responses [71–73]. Some experts argue that microglial progenitors in the bone marrow and tissue macrophages may therefore be equally affected and that secondary effects are elicited by employing CSF-1R inhibitors [56, 72]. Another disadvantage using CSF-1R inhibitor during neurodevelopment is that microglial death may alter neuronal activity and synaptic physiology [74], and so these potential adverse effects on development must be considered for clinical applications of microglial depletion. Further studies are still needed to specifically ablate microglia in the brain (using GW2580) with little effects on other tissues.

Liposomal clodronate injection can be regarded as another method to specifically deplete microglia. Hippocampal injection of clodronate provides specific anatomical depletion of Iba-1^+^ microglia, leading to alterations in spatial learning and sociability [75].

In addition, the timing of microglia depletion is another important factor that needs further investigation. Previous studies have indicated that there were no obvious behavioral consequences in adult mice following microglial depletion, despite extensive neuronal loss in the brain [51]. In contrast, microglia depletion in early life using liposomal clodronate can lead to increased locomotion and decreased anxiety and despair behaviors throughout life, confirming that microglia are important in facilitating proper brain development [76, 77].

## Genetic manipulation of microglia depletion

Although depletion of microglia can be achieved in mice carrying gene mutations that are important for the survival and development of microglia, mice can suffer from severe developmental defects and rarely survive into adulthood [45, 56]. With the advent of novel genetic manipulation methods, microglia ablation is more accessible and specific than pharmacological strategies (Table 2). The main advantages of genetic microglial depletion using cell type promoters that are coupled with suicide genes are that they have few side-effects on other peripheral tissues and that the efficiency is relatively higher than pharmacological approaches [72].

**Table 2:** Overview of genetic microglia depletion studies

Depletion strategy	Efficiency	Time window	Physiological effects	References
CD11b-HSVTK	56%	7 days	little effect on axonal degeneration in concussive brain injury	[80]
CD11b-HSVTK	>90%	7 days	represses EAE-associated neuroinflammation	[81]
CD11b-HSVTK	>90%	2 weeks	circulating monocytes occupy brain after microglia depletion	[79]
CD11b-HSVTK^mt-30^	51%	30 days	has no effect on motor neuron degeneration in ALS	[82]
CX3CR1^CRE^ DTR	80%	3 days	causes a cytokine storm and astrogliosis	[85]
CX3CR1^CRE^ DTR	99%	1 day	reduces synaptic structural plasticity associated with learning	[84]

To achieve the depletion of microglia, transgenic mice that express the suicide gene herpes simplex virus thymidine kinase (HSVTK) and mutant form HSVTK^mt-30^ under the specific CD11b promoter were generated [78–80]. HSVTK is a commonly used suicide gene that can be activated and becomes toxic (inducing apoptosis and inhibition of DNA) after administration of the pro-drug ganciclovir with >90% microglia being depleted in CD11b-HSVTK transgenic mice [79]. Heppner et al. reported that elimination of microglia in these transgenic mice could repress neuroinflammation as well as clinical signs of EAE [81]. In addition, microglia ablation in mutant form HSV-1 TK^mt-30^ mice had little effect on motor neuron degeneration in the context of amyotrophic lateral sclerosis (ALS), showing that microglia-expressing mutant genes may not contribute to the neurodegenerative process in ALS [78, 82, 83].

As an alternative genetic approach, diphtheria toxin (DT) is another widely used immunotoxin to deplete many cell types [72]. Recently, tamoxifen-induced CX3CR1^CreER^ mouse models that drive diphtheria toxin receptor (DTR) expression upon Cre-mediated recombination were established to specifically deplete microglia from the brain by systemic administration of DT [84, 85]. The efficiency of microglia depletion can reach ~90% and CX3CR1^+^ cells in the periphery remained unaffected using this method [84–86]. Microglia depletion in CX3CR1^CreER^ mice significantly reduced synaptic structural plasticity associated with multiple learning tasks via brain-derived neurotrophic factor signaling [84]. Moreover, specific and massive depletion of microglia can cause a rapid repopulation from novel internal pools expressing the stem cell marker nestin^+^ via IL-1 signaling without the contribution from peripherally circulating cells [85]. Previous studies of genetic microglial depletion have reported a rapid repopulation within 5 days, but these new cells are not fully characterized as yet [72].

## Concluding remarks and future directions

Despite remarkable major recent advances in our understanding of microglial biology regarding origins, development, phenotypes and functions over the past century, we are just beginning to decipher the enigmatic nature of these versatile players in order to best harness or modulate them in CNS disorders. Thanks to novel and cutting-edge manipulation tools to investigate microglia we are much closer to achieving these goals for therapeutic interventions. Herein we have discussed several microglia depletion systems including genetic targeting and pharmacological therapies. Among these, oral drug strategies may equally affect other cell types expressing the targeted receptors in the periphery, generally suppressing all immune response and eliciting secondary effects. For this reason, clinical translation of pharmacological strategies should be considered with caution.

Looking to the future, we would suggest further investigation of the relative importance of microglial subpopulation depletion at different disease stages. Only when we are sufficiently knowledgeable should we consider using microglia depletion as a clinical intervention to resolve neuroinflammation and to promote recovery. It would also be vital to take advantage of knowledge learned from microglia-specific genetic tools such as the tamoxifen-inducible CX3CR1^CreER^ mice and other microglia-specific knockdown strategies.

Finally, the relative merits of microglial depletion contra microglial functional inhibition should be thoroughly investigated, as the latter should theoretically allow for microglial functional repolarization in vivo, even if this has proven difficult in vitro. A prerequisite for this will be to ascertain whether the published reports of microglial depletion actually reflect inhibition instead with associated down-regulation of microglial surface receptor expression, and this should be re-analysed using the newest described microglia-specific markers. One caveat with all potential clinical applications of microglial depletion will be whether the myeloid cells repopulating the empty niche in the CNS, be they fast-proliferating microglia or infiltrating peripheral monocytes, will assume the full functionality of homeostatic CNS microglia, and this will be a critical issue to address. In conclusion, with improved and intricate cell type-specific targeting approaches, we are optimistic that future research will bear exciting new discoveries in this field.

## Abbreviations

CNS: Central nervous system
CSF1R: Colony-stimulating factor receptor
DT: Diphtheria toxin
DTR: Diphtheria toxin receptor
EAE: Experimental autoimmune encephalomyelitis
EMPs: Erythromyeloid progenitors
HSCs: Hematopoietic stem cells
HSVTK: Herpes simplex virus thymidine kinase
Iba-1: Ionized binding adaptor molecule-1
IL: Interleukin
LPS: Lipopolysaccharide
MRC-1: Mannose receptor C type 1
TGF-β: Transforming growth factor-β
Tmem119: Transmembrane protein 119
TNF-α: Tumor necrosis factor-α

## References

[1] Sierra A, de Castro F, Del Rio-Hortega J, Rafael Iglesias-Rozas J, Garrosa M, Kettenmann H (2016). The "Big-Bang" for modern glial biology: translation and comments on Pio del Rio-Hortega 1919 series of papers on microglia. Glia.

[2] Wolf SA, Boddeke HW, Kettenmann H. Microglia in physiology and disease. Annu Rev Physiol. 2016; 10.1146/annurev-physiol-022516-034406 27959620

[3] Masuda T, Prinz M (2016). Microglia: a unique versatile cell in the central nervous system. ACS Chem Neurosci.

[4] Benarroch EE (2013). Microglia: Multiple roles in surveillance, circuit shaping, and response to injury. Neurology.

[5] Grubman A, Kanninen KM, Malm T (2016). Multitasking Microglia and Alzheimer's disease: diversity, tools and therapeutic targets. J Mol Neurosci.

[6] Du L, Zhang Y, Chen Y, Zhu J, Yang Y, Zhang HL. Role of microglia in neurological disorders and their potentials as a therapeutic target. Mol Neurobiol. 2016; 10.1007/s12035-016-0245-0 27830532

[7] Guruswamy R, El Ali A. Complex roles of microglial cells in ischemic stroke pathobiology: new insights and future directions. Int J Mol Sci. 2017:18. 10.3390/ijms18030496 PMC5372512 28245599

[8] Eyo UB, Murugan M, Wu LJ (2017). Microglia-neuron communication in epilepsy. Glia.

[9] Hambardzumyan D, Gutmann DH, Kettenmann H (2016). The role of microglia and macrophages in glioma maintenance and progression. Nat Neurosci.

[10] Rodriguez MJ, Mahy N (2016). Neuron-microglia interactions in motor neuron degeneration. The inflammatory hypothesis in amyotrophic lateral sclerosis revisited. Curr Med Chem.

[11] Le W, Wu J, Tang Y (2016). Protective microglia and their regulation in Parkinson's disease. Front Mol Neurosci.

[12] Zhao H, Alam A, Chen Q, AE M, Pal A, Eguchi S (2017). The role of microglia in the pathobiology of neuropathic pain development: what do we know?. Br J Anaesth.

[13] Peng J, Gu N, Zhou L, BE U, Murugan M, Gan WB (2016). Microglia and monocytes synergistically promote the transition from acute to chronic pain after nerve injury. Nat Commun.

[14] Michell-Robinson MA, Touil H, Healy LM, Owen DR, Durafourt BA, Bar-Or A, et al. Roles of microglia in brain development, tissue maintenance and repair. Brain. 2015;138:1138-1159. 10.1093/brain/awv066 PMC5963417 25823474

[15] Mosser CA, Baptista S, Arnoux I, Audinat E. Microglia in CNS development: shaping the brain for the future. Progress in neurobiology. 2017; 10.1016/j.pneurobio.2017.01.002 28143732

[16] Jin X, Yamashita T (2016). Microglia in central nervous system repair after injury. J Biochem.

[17] Colonna M, Butovsky O. Microglia function in the central nervous system during health and neurodegeneration. Annu Rev Immunol. 2017; 10.1146/annurev-immunol-051116-052358 PMC8167938 28226226

[18] Thompson KK, Tsirka SE. The diverse roles of microglia in the neurodegenerative aspects of Central Nervous System (CNS) autoimmunity. Int J Mol Sci. 2017;18 10.3390/ijms18030504 PMC5372520 28245617

[19] Shemer A, Erny D, Jung S, Prinz M (2015). Microglia plasticity during health and disease: an immunological perspective. Trends Immunol.

[20] Schafer DP, Stevens B (2015). Microglia function in central nervous system development and plasticity. Cold Spring Harb Perspect Biol.

[21] Bilimoria PM, Stevens B (2015). Microglia function during brain development: new insights from animal models. Brain Res.

[22] Reemst K, Noctor SC, Lucassen PJ, Hol EM (2016). The inedispensable roles of microglia and astrocytes during brain development. Front Hum Neurosci.

[23] Hong S, Dissing-Olesen L, Stevens B (2016). New insights on the role of microglia in synaptic pruning in health and disease. Curr Opin Neurobiol.

[24] Frost JL, Schafer DP (2016). Microglia: architects of the developing nervous system. Trends Cell Biol.

[25] Kettenmann H, Kirchhoff F, Verkhratsky A (2013). Microglia: new roles for the synaptic stripper. Neuron.

[26] Tang Y, Le W (2016). Differential roles of M1 and M2 microglia in neurodegenerative diseases. Mol Neurobiol.

[27] Fan X, Zhang H, Cheng Y, Jiang X, Zhu J, Jin T (2016). Double roles of macrophages in human neuroimmune diseases and their animal models. Mediat Inflamm.

[28] Bogie JF, Stinissen P, Hendriks JJ (2014). Macrophage subsets and microglia in multiple sclerosis. Acta Neuropathol.

[29] Cartier N, Lewis CA, Zhang R, Rossi FM (2014). The role of microglia in human disease: therapeutic tool or target?. Acta Neuropathol.

[30] Biber K, Moller T, Boddeke E, Prinz M (2016). Central nervous system myeloid cells as drug targets: current status and translational challenges. Nat Rev Drug Discov.

[31] Zhang XM, Lund H, Mia S, Parsa R, Harris RA (2014). Adoptive transfer of cytokine-induced immunomodulatory adult microglia attenuates experimental autoimmune encephalomyelitis in DBA/1 mice. Glia.

[32] Mia S, Warnecke A, Zhang XM, Malmstrom V, Harris RA (2014). An optimized protocol for human M2 macrophages using M-CSF and IL-4/IL-10/TGF-beta yields a dominant immunosuppressive phenotype. Scand J Immunol.

[33] Ginhoux F, Prinz M (2015). Origin of microglia: current concepts and past controversies. Cold Spring Harb Perspect Biol.

[34] Mildner A, Schmidt H, Nitsche M, Merkler D, Hanisch UK, Mack M (2007). Microglia in the adult brain arise from Ly-6ChiCCR2+ monocytes only under defined host conditions. Nat Neurosci.

[35] Sheng J, Ruedl C, Karjalainen K (2015). Most tissue-resident macrophages except microglia are derived from fetal hematopoietic stem cells. Immunity.

[36] Crotti A, Ransohoff RM (2016). Microglial physiology and pathophysiology: insights from genome-wide transcriptional profiling. Immunity.

[37] Ginhoux F, Greter M, Leboeuf M, Nandi S, See P, Gokhan S (2010). Fate mapping analysis reveals that adult microglia derive from primitive macrophages. Science.

[38] Tay TL, Hagemeyer N, Prinz M (2016). The force awakens: insights into the origin and formation of microglia. Curr Opin Neurobiol.

[39] Prinz M, Priller J (2014). Microglia and brain macrophages in the molecular age: from origin to neuropsychiatric disease. Nat Rev Neurosci.

[40] Prinz M, Erny D, Hagemeyer N (2017). Ontogeny and homeostasis of CNS myeloid cells. Nat Immunol.

[41] Askew K, Gomez-Nicola D. A story of birth and death: Insights into the formation and dynamics of the microglial population. Brain Behav Immun. 2017; 10.1016/j.bbi.2017.03.009 28341583

[42] Gomez Perdiguero E, Klapproth K, Schulz C, Busch K, Azzoni E, Crozet L (2015). Tissue-resident macrophages originate from yolk-sac-derived erythro-myeloid progenitors. Nature.

[43] Derecki NC, Katzmarski N, Kipnis J, Meyer-Luehmann M (2014). Microglia as a critical player in both developmental and late-life CNS pathologies. Acta Neuropathol.

[44] De I, Nikodemova M, Steffen MD, Sokn E, Maklakova VI, Watters JJ (2014). CSF1 overexpression has pleiotropic effects on microglia in vivo. Glia.

[45] Butovsky O, Jedrychowski MP, Moore CS, Cialic R, Lanser AJ, Gabriely G (2014). Identification of a unique TGF-beta-dependent molecular and functional signature in microglia. Nat Neurosci.

[46] Muffat J, Li Y, Yuan B, Mitalipova M, Omer A, Corcoran S (2016). Efficient derivation of microglia-like cells from human pluripotent stem cells. Nat Med.

[47] Hammond TR, Stevens B (2016). Increasing the neurological-disease toolbox using iPSC-derived microglia. Nat Med.

[48] Xu J, Zhu L, He S, Wu Y, Jin W, Yu T (2015). Temporal-spatial resolution fate mapping reveals distinct origins for embryonic and adult microglia in zebrafish. Dev Cell.

[49] Askew K, Li K, Olmos-Alonso A, Garcia-Moreno F, Liang Y, Richardson P (2017). Coupled proliferation and apoptosis maintain the rapid turnover of microglia in the adult brain. Cell Rep.

[50] Hughes EG, Bergles DE (2014). Hidden progenitors replace microglia in the adult brain. Neuron.

[51] Elmore MR, Najafi AR, Koike MA, Dagher NN, Spangenberg EE, Rice RA (2014). Colony-stimulating factor 1 receptor signaling is necessary for microglia viability, unmasking a microglia progenitor cell in the adult brain. Neuron.

[52] Bennett ML, Bennett FC, Liddelow SA, Ajami B, Zamanian JL, Fernhoff NB (2016). New tools for studying microglia in the mouse and human CNS. Proc Natl Acad Sci U S A.

[53] Wohleb ES, Delpech JC. Dynamic cross-talk between microglia and peripheral monocytes underlies stress-induced neuroinflammation and behavioral consequences. Prog Neuro-Psychopharmacol Biol Psychiatry. 2016; 10.1016/j.pnpbp.2016.04.013 27154755

[54] Sousa C, Biber K, Michelucci A (2017). Cellular and molecular characterization of microglia: a unique immune cell population. Front Immunol.

[55] Glass R, Synowitz M (2014). CNS macrophages and peripheral myeloid cells in brain tumours. Acta Neuropathol.

[56] Waisman A, Ginhoux F, Greter M, Bruttger J (2015). Homeostasis of microglia in the adult brain: review of novel microglia depletion systems. Trends Immunol.

[57] Buttgereit A, Lelios I, Yu X, Vrohlings M, Krakoski NR, Gautier EL (2016). Sall1 is a transcriptional regulator defining microglia identity and function. Nat Immunol.

[58] Satoh J, Kino Y, Asahina N, Takitani M, Miyoshi J, Ishida T (2016). TMEM119 marks a subset of microglia in the human brain. Neuropathology.

[59] Mills CD, Harris RA, Ley K. Macrophage polarization: decisions that affect health. Journal of clinical & cellular immunology. 2015;6 10.4172/2155-9899.1000364 PMC4780849 26962469

[60] Harris RA (2014). Spatial, temporal, and functional aspects of macrophages during "The Good, the Bad, and the Ugly" phases of inflammation. Front Immunol.

[61] Parisi C, Napoli G, Pelegrin P, Volonte C (2016). M1 and M2 functional imprinting of primary microglia: role of P2X7 activation and miR-125b. Mediat Inflamm.

[62] Pelegrin P, Surprenant A (2009). Dynamics of macrophage polarization reveal new mechanism to inhibit IL-1beta release through pyrophosphates. EMBO J.

[63] Elmore MR, Lee RJ, West BL, Green KN (2015). Characterizing newly repopulated microglia in the adult mouse: impacts on animal behavior, cell morphology, and neuroinflammation. PLoS One.

[64] Rice RA, Pham J, Lee RJ, Najafi AR, West BL, Green KN. Microglial repopulation resolves inflammation and promotes brain recovery after injury. Glia. 2017; 10.1002/glia.23135 PMC5395311 28251674

[65] Spangenberg EE, Lee RJ, Najafi AR, Rice RA, Elmore MR, Blurton-Jones M (2016). Eliminating microglia in Alzheimer's mice prevents neuronal loss without modulating amyloid-beta pathology. Brain J Neurol.

[66] Li M, Li Z, Ren H, Jin WN, Wood K, Liu Q, et al. Colony stimulating factor 1 receptor inhibition eliminates microglia and attenuates brain injury after intracerebral hemorrhage. J Cereb Blood Flow Metab. 2016; 10.1177/0271678X16666551 PMC5482387 27596835

[67] Szalay G, Martinecz B, Lenart N, Kornyei Z, Orsolits B, Judak L (2016). Microglia protect against brain injury and their selective elimination dysregulates neuronal network activity after stroke. Nat Commun.

[68] Yao Y, Echeverry S, Shi XQ, Yang M, Yang QZ, Wang GY (2016). Dynamics of spinal microglia repopulation following an acute depletion. Sci Rep.

[69] Jin WN, Shi SX, Li Z, Li M, Wood K, Gonzales RJ, et al. Depletion of microglia exacerbates postischemic inflammation and brain injury. J Cereb Blood Flow Metab. 2017:271678X17694185. 10.1177/0271678X17694185 PMC5444553 28273719

[70] Acharya MM, Green KN, Allen BD, Najafi AR, Syage A, Minasyan H (2016). Elimination of microglia improves cognitive function following cranial irradiation. Sci Rep.

[71] Chalmers SA, Wen J, Shum J, Doerner J, Herlitz L, Putterman C. CSF-1R inhibition attenuates renal and neuropsychiatric disease in murine lupus. Clin Immunol. 2016; 10.1016/j.clim.2016.08.019 PMC5326697 27570219

[72] Jakel S, Dimou L (2017). Glial cells and their function in the adult brain: a journey through the history of their ablation. Front Cell Neurosci.

[73] Crespo O, Kang SC, Daneman R, Lindstrom TM, Ho PP, Sobel RA (2011). Tyrosine kinase inhibitors ameliorate autoimmune encephalomyelitis in a mouse model of multiple sclerosis. J Clin Immunol.

[74] Eyo UB, Wu LJ (2013). Bidirectional microglia-neuron communication in the healthy brain. Neural Plast.

[75] Torres L, Danver J, Ji K, Miyauchi JT, Chen D, Anderson ME (2016). Dynamic microglial modulation of spatial learning and social behavior. Brain Behav Immun.

[76] Nelson LH, Lenz KM (2017). Microglia depletion in early life programs persistent changes in social, mood-related, and locomotor behavior in male and female rats. Behav Brain Res.

[77] VanRyzin JW, Yu SJ, Perez-Pouchoulen M, McCarthy MM. Temporary depletion of microglia during the early postnatal period induces lasting sex-dependent and sex-independent effects on behavior in rats. eNeuro. 2016;3 10.1523/ENEURO.0297-16.2016 PMC5144556 27957532

[78] Gowing G, Vallieres L, Julien JP (2006). Mouse model for ablation of proliferating microglia in acute CNS injuries. Glia.

[79] Varvel NH, Grathwohl SA, Baumann F, Liebig C, Bosch A, Brawek B (2012). Microglial repopulation model reveals a robust homeostatic process for replacing CNS myeloid cells. Proc Natl Acad Sci U S A.

[80] Bennett RE, Brody DL (2014). Acute reduction of microglia does not alter axonal injury in a mouse model of repetitive concussive traumatic brain injury. J Neurotrauma.

[81] Heppner FL, Greter M, Marino D, Falsig J, Raivich G, Hovelmeyer N (2005). Experimental autoimmune encephalomyelitis repressed by microglial paralysis. Nat Med.

[82] Gowing G, Philips T, Van Wijmeersch B, Audet JN, Dewil M, Van Den Bosch L (2008). Ablation of proliferating microglia does not affect motor neuron degeneration in amyotrophic lateral sclerosis caused by mutant superoxide dismutase. J Neurosci.

[83] Wieghofer P, Knobeloch KP, Prinz M (2015). Genetic targeting of microglia. Glia.

[84] Parkhurst CN, Yang G, Ninan I, Savas JN, Yates JR, Lafaille JJ (2013). Microglia promote learning-dependent synapse formation through brain-derived neurotrophic factor. Cell.

[85] Bruttger J, Karram K, Wortge S, Regen T, Marini F, Hoppmann N (2015). Genetic cell ablation reveals clusters of local self-renewing microglia in the mammalian central nervous system. Immunity.

[86] Wieghofer P, Prinz M (1862). Genetic manipulation of microglia during brain development and disease. Biochim Biophys Acta.

